# Extended spectrum β-lactamase-producing *Escherichia coli* forms filaments as an initial response to cefotaxime treatment

**DOI:** 10.1186/s12866-015-0399-3

**Published:** 2015-03-08

**Authors:** Thea SB Kjeldsen, Morten OA Sommer, John E Olsen

**Affiliations:** Department of Veterinary Disease Biology, Faculty of Health and Medical Sciences, University of Copenhagen, 1870 Frederiksberg C, Denmark; Department of Systems Biology, Technical University of Denmark, 2800 Lyngby, Denmark; Novo Nordisk Foundation Center for Biosustainability, Technical University of Denmark, 2970 Hørsholm, Denmark

**Keywords:** Antimicrobial resistance, CTX-M-1, Filaments

## Abstract

**Background:**

β-lactams target the peptidoglycan layer in the bacterial cell wall and most β-lactam antibiotics cause filamentation in susceptible Gram-negative bacteria at low concentrations. The objective was to determine the initial morphological response of cephalosporin resistant CTX-M-1-producing *E. coli* to cefotaxime and to determine whether the response depended on the growth phase of the bacterium and the concentration of antibiotic.

**Results:**

Two antibiotic resistant strains carrying *bla*_CTX-M-1_ on the chromosome and on an IncI1 plasmid and three sensitive strains were used in this study. The resistant strains displayed elongated cells when exposed to cefotaxime at sub-inhibitory as well as therapeutic concentrations (1 to 512 mg/L of cefotaxime) in both lag and early exponential phase, suggesting that the elongation was an initial response mechanism to the antibiotic. Normal sized cells were the dominant cell type in exponential and stationary growth phase. No elongated cells were seen in cultures without cefotaxime. In cultures with high concentrations of cefotaxime (128–512 mg/L), no growth other than initial filamentation was observed, but spheroplats appeared after 14–17 hours in cultures of the resistant strains. Filaments were also observed in sensitive control strains with sub-inhibitory concentrations of cefotaxime.

**Conclusions:**

We showed that *E. coli* resistant to β-lactams by an extended-spectrum β-lactamase, *bla*_CTX-M-1_, produced filaments when exposed to cefotaxime. The filament formation was restricted to early growth phases and the time the cells grew as filaments was antibiotic concentration dependent. This indicates that antibiotic resistant *E. coli* undergo the same morphological changes as sensitive bacteria in the presence of β-lactam antibiotic. It was showed that the filament formation was an initial response to the antibiotics.

## Background

β-lactams are bactericidal antibiotics that target the peptidoglycan layer in the bacterial cell wall by covalently binding to the penicillin-binding proteins. This inhibits the last stage of peptidoglycan synthesis, namely the crosslinking formation [1,2]. Gram-negative bacteria generally respond to β-lactam antibiotics by either (a) rapid cell lysis, (b) the production of cell-wall-deficient round cells or (c) filament formation [3]. Most β-lactam antibiotics cause filamentation in susceptible Gram-negative bacteria at low concentrations [3,4]. These morphology changes often depend on the antibiotic concentration and the binding site specifications [3,5]. Greenwood *et al.* found that cephalosporins, such as cefotaxime, induce filament production from susceptible *Escherichia coli* up to a concentration of 2 mg/L [6]. However, only limited knowledge is available about morphology changes in β-lactam resistant bacteria treated with β-lactams. Demirel *et al.* recently found that extended spectrum β-lactamase producing uropathogenic *E. coli* changed their morphology into filamentous forms when treated with ceftibuten [7]. However, the bacteria did not change morphology when treated with trimethoprim or ciprofloxacin.

In this study, we investigated the morphology changes of extended spectrum β-lactamase-producing *E. coli* (carrying *bla*_CTX-M-1_) exposed to cefotaxime at sub-inhibitory and therapeutic concentrations. The objectives of this study were i) to determine if an antibiotic resistant CTX-M-1-producing *E. coli* exposed to cefotaxime at sub-inhibitory and therapeutic concentrations produced filaments, even though the strain is resistant to β-lactams and ii) to evaluate if the filamentation was growth phase and antibiotic concentration dependent.

## Methods

### Bacterial strains

The bacteria used in this study carried *bla*_CTX-M-1_ on the chromosome (MG1655/CTX-M-1 (*E. coli* K-12 MG1655 ∆YbeM::CTX-M-1 )) and on an IncI1 plasmid (MG1655/IncI1/CTX-M-1 (E. coli K-12 MG1655 + IncI1/CTX-M-1)). The strains have previously been described in detail [8]. Two control strains, MG1655/NCS and MG1655/IncI1/NCS containing a non-coding sequence (NCS) in the chromosome and on an IncI1 plasmid, respectively, with the same length as *bla*_CTX-M-1_ were constructed to control for insertion site and plasmid. The NCS was cloned into the pseudo gene *ybeM* [9] of *E. coli* K-12 MG1655 using the Lambda Red recombination system as described previously [10-12]. Plasmid p2795 was used to construct template vectors. Sequences of oligonucleotides used for Lambda Red mediated mutagenesis and PCR verifications were: NCS-ybeM-F: 5′- ATGCTGGTGGCACTTCAGGCAGGAAACATCGTCGCCCGTAATTACGCATGATTATAATGCGTCAGCC-3′, NCS-ybeM-R: 5′-AGGCGGCAGGAAGTACCAGGATTTCAGCTCCCTGTAATCGTGTAGGCTGGAGCTGCTTC-3′, NCS-IncI1-F: 5′-CACACGTGGAATTTAGGTTAGACTATAAATAGAAAAATTACGCATGATTATAATGCGTCAGCC-3′, IncI1-R: 5′-TCTAAGGCGATAAACAAAAACGGAATGAGTTTCCCCATTCGTGTAGGCTGGAGCTGCTTC-3′, NCS-F: 5′-GATTATAATGCGTCAGCCCGTGTAGG-3′ and NCS-R: 5′-GATAGTAGTCCGCCCATTCGAACGG-3′. Insertions were confirmed by PCR and sequencing using standard procedures. Strains were maintained in Difco™ Lysogeny broth (LB), Lennox (Becton, Dickinson and Company, Albertslund, Denmark) and on LB agar plates (Becton, Dickinson and Company, Albertslund, Denmark). During strain construction the medium was supplemented with kanamycin (30 mg/L) (Sigma, Copenhagen, Denmark) when appropriate.

### Growth conditions

Bacterial morphology was investigated by performing growth experiments on an oCelloScope (Unisensor, Alleroed, Denmark) for 24 hours at 37°C (no shaking). Cultures were prepared in aliquots of 100 μL Mueller Hinton II (MH-2) broth (Sigma, Copenhagen, Denmark) supplemented with 0 to 512 mg/L of cefotaxime (in two-fold dilutions) (Sigma, Copenhagen, Denmark) for the resistant strains and supplemented with 0 to 4 mg/L of cefotaxime (in two-fold dilutions) for the sensitive strains. The cultures were inoculated with 1–5 single colonies from a blood agar plate (blood agar base (Oxoid, Roskilde, Denmark) supplemented with 5% blood from cattle) to a final cell density of 10^6^ cfu/mL, using a Sensititre™ Nephelometer (Thermo Scientific™, Roskilde, Denmark) with a McFarland 0.5 standard (1–2 x 10^8^ cfu/mL). Cefotaxime was added to the media just before inoculation with cells from blood plates. Images of the cultures were acquired with 10-minute intervals through the oCelloScope bright field camera (magnification of approximately 200× and resolution of 1.3 μm). The growth of the cultures was also measured by the oCelloScope using a background corrected absorption algorithm (BCA, former called pixel histogram summation (PHS)) [13]. For more detailed pictures culture material was investigated in a Zeiss microscope with a ph3 Plan-neoflur 100x/1.30 oli, ∞/1.7 lens with a Zeiss AxioCam camera.

### Antimicrobial susceptibility testing

The MIC of cefotaxime was determined using the broth microdilution method following the CLSI guidelines [14], as described previously [8].

## Results

The MIC of cefotaxime for the two isogenic β-lactam resistant strains, MG1655/CTX-M-1 carrying *bla*_CTX-M-1_ on the chromosome and MG1655/IncI1/CTX-M-1 carrying *bla*_CTX-M-1_ on an IncI1 plasmid have been determined in a previous study and were 168 mg/L ± 4 mg/L and 252 mg/L ± 4 mg/L, respectively [8]. The MICs of cefotaxime were 0.032 mg/L, 0.032 mg/L and 0.064 mg/L for the three sensitive control strains *E. coli* K-12 MG1655, MG1655/NCS, and MG1655/IncI1/NCS, respectively.

**Figure 1:**
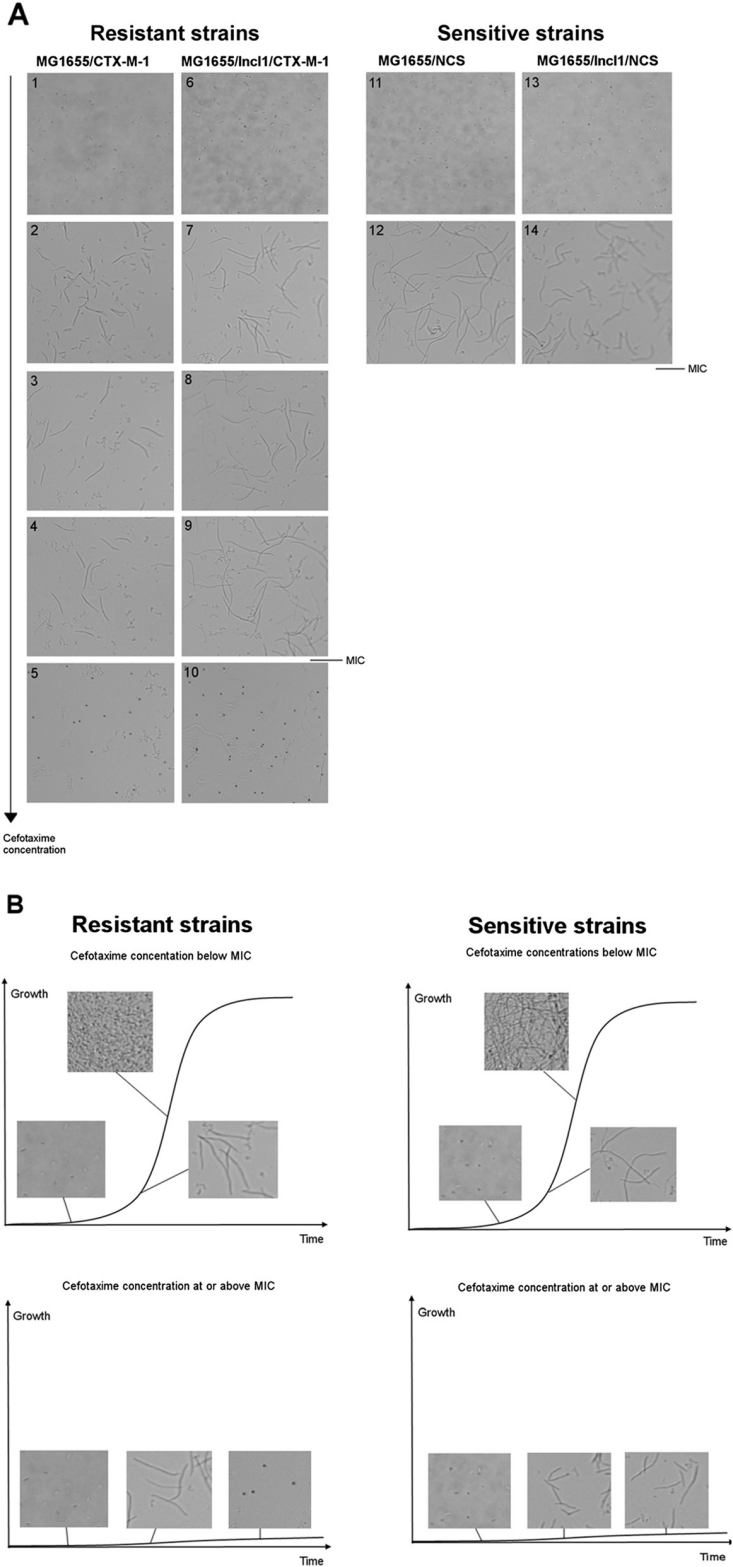
**Cell morphology of MG1655/CTX-M-1, MG1655/IncI1/CTX-M-1, MG1655/NCS and MG1655/IncI1/NCS cultured with and without cefotaxime. (A)** Filmentation at different cefotaxime concentrations. Pictures are taken in early exponential phase, except where stated otherwise. (1–5) MG1655/CTX-M-1 cultured without cefotaxime, with 4 mg/L cefotaxime, with 16 mg/L cefotaxime, with 32 mg/L cefotaxime and with 256 mg/L cefotaxime, respectively. (6–10) MG1655/IncI1/CTX-M-1 cultured without cefotaxime, with 4 mg/L cefotaxime, with 16 mg/L cefotaxime, with 64 mg/L cefotaxime and with 256 mg/L cefotaxime, respectively. Picture (5) and (10) were taken in late stationary phase. (11–12) MG1655/NCS cultured without cefotaxime and with 0.016 mg/L cefotaxime, respectively. (13–14) MG1655/IncI1/NCS cultured without cefotaxime and with 0.032 mg/L cefotaxime, respectively. **(B)** Filamentation and growth phase dependency. The growth curves are representative drawings, where the morphology of the cells (resistant or sensitive) is marked at different growth phases. The strains were grown in MH-2 broth with and without cefotaxime on an oCelloScope.

**Figure 2:**
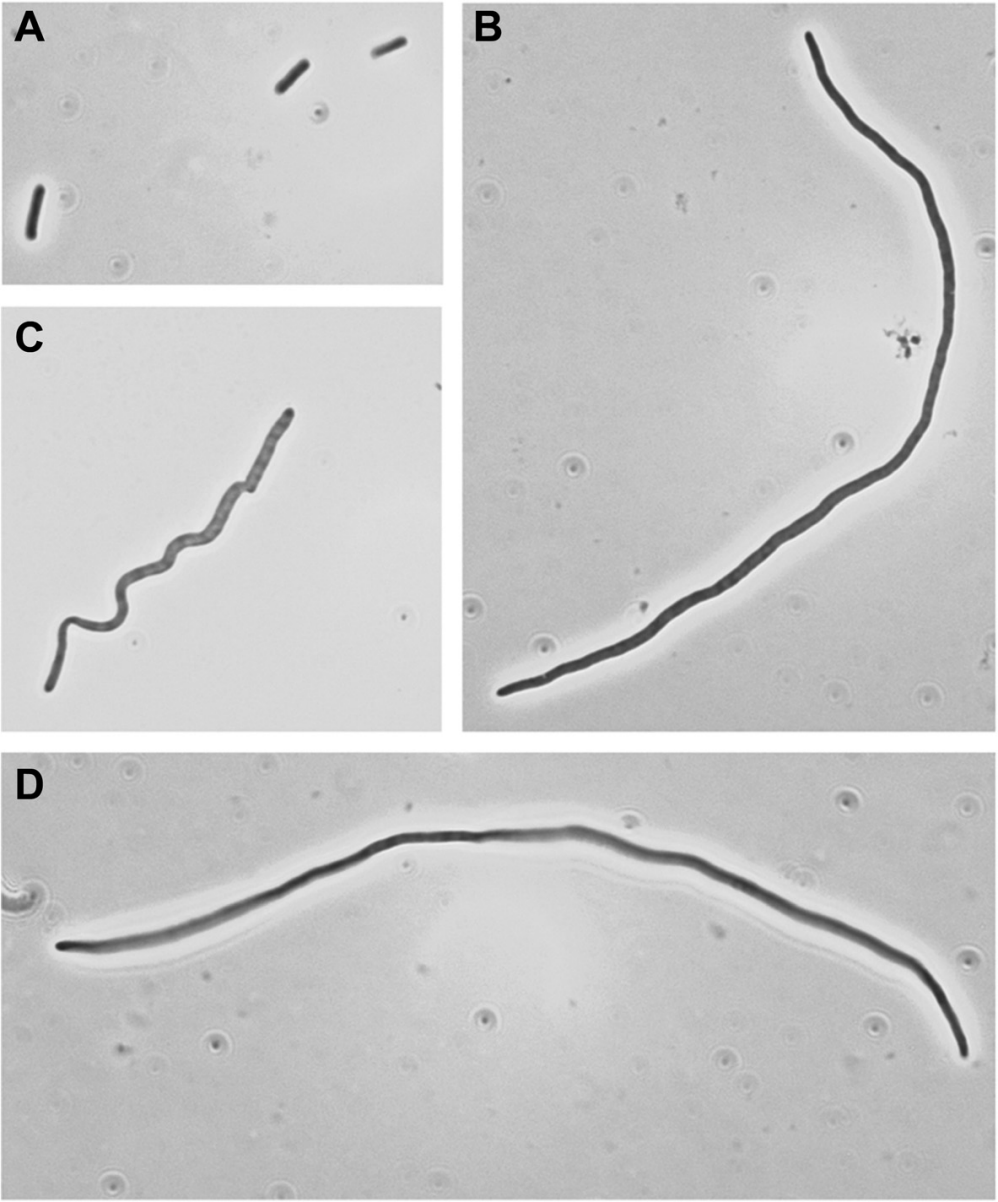
**Cell morphology of MG1655/CTX-M-1 and MG1655/IncI1/CTX-M-1 cultured with and without cefotaxime.** Pictures are taken in early exponential phase. **(A)** MG1655/IncI1/CTX-M-1 cultured without cefotaxime, **(B)** MG1655/CTX-M-1 cultured with 32 mg/L cefotaxime, **(C)** MG1655/CTX-M-1 cultured with 2 mg/L cefotaxime and **(D)** MG1655/IncI1/CTX-M-1 cultured with 16 mg/L cefotaxime. MG1655/CTX-M-1 and MG1655/IncI1/CTX-M-1 were grown in MH-2 broth with and without cefotaxime and pictures were taken with a Zeiss microscope.

Normal sized growing cells were observed in the cultures of cefotaxime-resistant MG1655/CTX-M-1 and MG1655/IncI1/CTX-M-1 when grown without cefotaxime (Figure 1A-1, 6). However, filaments were observed in all cultures with 1 mg/L to 512 mg/L of cefotaxime already in the lag and early exponential growth phase (Figure 1A-2, 4, 5, 7, 8, 9, 10 and Figure 2), and the filaments remained the predominant cell format, and right after onset to exponential phase (here defined according to growth measured on an oCelloScope by BCA; lag phase: approx. 0–0.1, early exponential phase: approx. 0.1-0.4, exponential phase: approx. 0.4-2.2 and stationary phase: approx. 2.2-2.5). In cultures of the resistant strains with concentrations of cefotaxime below the MIC, normal sized cells started to be seen together with elongated cells when the culture entered exponential phase (Figure 1B), and in exponential growth phase, this cell type was the dominant one (BCA approx. 0.6). The filaments were up to 16 times longer than normal sized cells (Figure 2). In cultures with cefotaxime at or above the MIC of the two resistant strains, elongated cells formed and were present 2 hours after inoculation even though no further growth was seen in the culture (Figure 1B). In these cultures, spheroplats were observed after 14–17 hours (Figure 1A-5, 10).

Filaments were not observed in the sensitive strains when grown without cefotaxime (Figure 1A-11, 13). When sub-inhibitory concentrations of cefotaxime were present, filaments were observed 2 hours after inoculation (Figure 1A-12, 14). In contrast to the resistant strains, growth continued in filament networks (Figure 1B). No growth was seen above 0.016 mg/L cefotaxime for MG1655 and MG1655/NCS and above 0.032 mg/L cefotaxime for MG1655/IncI1/NCS. In cultures with cefotaxime at or above the MIC of the strains, elongated cells were present 2 hours after inoculation even though no further growth was seen in the cultures (Figure 1B). Spheroplats were not seen in the cultures with the sensitive cells.

## Discussion

In this study we investigated the morphology changes of CTX-M-1-producing *E. coli* exposed to cefotaxime at sub-MIC, MIC and above MIC concentrations. Paulander *et al.* have previously demonstrated filamentation of wild-type *E. coli* MG1655 cells when exposed to ampicillin (5 g/L) and norfloxacin (250 ng/mL and 2500 ng/mL), when the cultures reached a cell density of 10^8^ cells/ml [15]. This corresponds well to the morphological response observed in the present study for cefotaxime-sensitive *E. coli* MG1655 cells, where filament formation was found to be the response to cefotaxime at both sub-MIC and MIC concentrations. Other studies also demonstrated that filament production occurs in sensitive Gram-negative strains [3,4]. In the current study we showed that *E. coli* resistant to β-lactams by an extended-spectrum β-lactamase, *bla*_CTX-M-1_, also produced filaments when exposed to cefotaxime. In a recent study performed by Demirel *et al.* it was shown that CTX-M-15-producing uropathogenic *E. coli* changed its morphology into filamentous forms when treated with ceftibuten [7]. These observations are in correlation with the ones observed in this study. The filament formation in this current study was observed as an initial growth response, irrespective of the antibiotic concentration, and filaments were observed in both lag and early logarithmic phase. When the cultures grew in the logarithmic phase normal size cell started to become dominant. Hence, the filamentation was restricted to certain growth phases and happened as an initial response to the cefotaxime treatment. Filamentation has been suggested to be involved in bacterial survival during antimicrobial therapies [16]. Hence, filamentation could be an intended response of the bacteria when it is stressed and threatened to be killed by antibiotics [16].

In the sensitive strains cultured with sub-MIC concentrations of cefotaxime, the growth continued in a filament network manner throughout the whole growth cycle, after the filaments were observed approximately 2 hours after the inoculation. Cefotaxime is active in the periplasm of *E. coli*, where it gets hydrolysed by the CTX-M-1 β-lactamase in the resistant strains. According to EcoCyc more than 35 cell division proteins exists. A study by Spartt showed that β-lactams that specifically inhibit cell division, bind preferentially to penicillin binding protein 3 (PBP3/ftsI) [17]. Cefotaxime shows high affinity for PBP3, which is required for septum formation. Studies have shown that when PBP3 is inhibited, the cells produce filaments [18,19]. Previous studies also showed that *sulA* (*sfiA*) and *capR* are involved in the filamentation of *E. coli* [20,21]. Furthermore, Miller *et al.* showed that the SOS-promoting *recA*, *lexA* and *dpiA* are involved in inhibiting cell division [22]. It is speculated that initially cefotaxime binds to PBP3 in the resistant strains, which induce the production of filaments. However, when CTX-M-1 was produced in high enough quantities in the resistant cells, cefotaxime was hydrolysed and not able to bind to PBP3 anymore. The cells then initiate growth with normal sized cells. In a previous study of ours it was shown that the CTX-M-1 protein levels increased 2 fold or more from lag to logarithmic phase [8], which suggests that it is not until logarithmic phase that high enough CTX-M-1 concentrations are present to degrade the drug. Furthermore, the time the cells grew as filament forms was antibiotic concentration dependent (longer lag phase with higher concentrations of drug [8]), which corresponds nicely with that it would take longer time for the CTX-M-1 enzyme to hydrolyse the drug “completely”, the higher the cefotaxime concentration, thus, prolonging the time before growth with normal sized cells can initiate. As the sensitive cells are not able to hydrolyse cefotaxime at all, they grow in filament forms in the entire growth cycle when sub-MIC concentrations of cefotaxime were present.

As mentioned, filaments were observed with all cefotaxime concentrations tested, which suggest that the filamentation formation *per se* was not concentration dependent. Elongated cells were developed and observed 2 hours after inoculation in cultures even with cefotaxime concentrations at or above the MIC. No further growth was observed in these cultures; however, the resistance strains did also not undergo further morphological changes for 14–17 hours and the sensitive cells did not undergo further morphological changes at all in the time investigated. This is very interesting as cefotaxime is a bactericidal drug, and more investigations are needed to fully understand this phenomenon and to demonstrate whether such cells have the ability to re-enter into a growing stage. Finally after 14–17 hours, spheroplats which are osmotically fragile cells [5] were observed in the cultures of the resistance strains at or above MIC. That fact that filaments were observed in the first 2 hours after inoculation at all cefotaxime concentrations, even those at or above MIC, suggests that it takes approximately 2–3 hours before the cefotaxime concentrations in the periplasm is high enough to fully be active. It has been shown that cefotaxime has a rather slow bacterial diffusion rate through porin-channels of *E. coli* K-12 [23], which may be a contributing factor to this observation.

## Conclusions

These results show that resistant *E. coli* undergoes the same morphological changes as sensitive *E. coli* in the presence of β-lactam antibiotic in the lag and initial logarithmic phase. However, when the β-lactams had been degraded to a critical concentration, the cells initiate growth with normal sized cells, in contrast to the sensitive strains, which continued growth in a filament network manner. The results suggest that the filamentation is an initial response to the cefotaxime treatment regardless of whether the bacteria are resistant or not.

## References

[1] Tipper DJ, Strominger JL (1965). Mechanism of action of penicillins: a proposal based on their structural similarity to acyl-D-alanyl-D-alanine. Proc Natl Acad Sci U S A.

[2] Yao Z, Kahne D, Kishony R (2012). Distinct single-cell morphological dynamics under beta-lactam antibiotics. Mol Cell.

[3] Gould IM, MacKenzie FM (1997). The response of Enterobacteriaceae to beta-lactam antibiotics-‘round forms, filaments and the root of all evil’. J Antimicrob Chemother.

[4] Hanberger H, Nilsson LE, Nilsson M, Maller R (1991). Postantibiotic effect of beta-lactam antibiotics on gram-negative bacteria in relation to morphology, initial killing and Mic. Eur J Clin Mircobiol.

[5] Greenwood D (1997). Antibiotic effects in vitro and the prediction of clinical response. J Antimicrob Chemother.

[6] Greenwood D, Pearson N, Eley A, O’Grady F (1980). Comparative *in vitro* activities of cefotaxime and ceftizoxime (FK749): new cephalosporins with exceptional potency. Antimicrob Agents Chemother.

[7] Demirel I, Kruse R, Önnberg A, Persson K (2015). Ceftibuten-induced filamentation of extended spectrum beta lactamase (ESBL)-producing uropathogenic *Escherichia coli* alters host cell responses during an in vitro infection. Microb Pathog.

[8] Kjeldsen TS, Overgaard M, Nielsen SS, Bortolaia V, Jelsbak L, Sommer M (2015). CTX-M-1 beta-lactamase expression in *Escherichia coli* is dependent on cefotaxime concentration, growth phase and gene location. J Antimicrob Chemother.

[9] Lerat E, Ochman H (2004). Psi-Phi: exploring the outer limits of bacterial pseudogenes. Genome Res.

[10] Datsenko KA, Wanner BL (2000). One-step inactivation of chromosomal genes in *Escherichia coli* K-12 using PCR products. Proc Natl Acad Sci U S A.

[11] Gerlach RG, Holzer SU, Jackel D, Hensel M (2007). Rapid engineering of bacterial reporter gene fusions by using Red recombination. Appl Environ Microbiol.

[12] Doublet B, Douard G, Targant H, Meunier D, Madec JY, Cloeckaert A (2008). Antibiotic marker modifications of lambda Red and FLP helper plasmids, pKD46 and pCP20, for inactivation of chromosomal genes using PCR products in multidrug-resistant strains. J Microbiol Methods.

[13] Fredborg M, Andersen KR, Jorgensen E, Droce A, Olesen T, Jensen BB (2013). Real-time optical antimicrobial susceptibility testing. J Clin Microbiol.

[14] CLSI: Clinical and Laboratory Standards Institute (2015). Performance Standards for Antimicrobial Susceptibility Testing: Twenty-First Informational Supplement M100-S25.

[15] Paulander W, Wang Y, Folkesson A, Charbon G, Lobner-Olesen A, Ingmer H (2014). Bactericidal antibiotics increase hydroxyphenyl fluorescein signal by altering cell morphology. PLoS One.

[16] Justice SS, Hunstad DA, Cegelski L, Hultgren SJ (2008). Morphological plasticity as a bacterial survival strategy. Nat Rev Microbiol.

[17] Spratt BG (1975). Distinct penicillin binding proteins involved in the division, elongation, and shape of *Escherichia coli* K12. Proc Natl Acad Sci.

[18] Selakovitch-Chenu L, Seroude L, Sicard AM (1993). The role of penicillin-binding protein 3 (PBP 3) in cefotaxime resistance in Streptococcus pneumoniae. Mol Gen Genet.

[19] Renggli S, Keck W, Jenal U, Ritz D (2013). Role of autofluorescence in flow cytometric analysis of *Escherichia coli* treated with bactericidal antibiotics. J Bacteriol.

[20] Schoemaker J, Gayda R, Markovitz A (1984). Regulation of cell division in *Escherichia coli*: SOS induction and cellular location of the sulA protein, a key to lon-associated filamentation and death. J Bacteriol.

[21] Mukherjee A, Cao C, Lutkenhaus J (1998). Inhibition of FtsZ polymerization by SulA, an inhibitor of septation in *Escherichia coli*. Proc Natl Acad Sci.

[22] Miller C, Thomsen LE, Gaggero C, Mosseri R, Ingmer H, Cohen SN (2004). SOS response induction by beta-lactams and bacterial defense against antibiotic lethality. Science.

[23] Yoshimura F, Nikaido H (1985). Diffusion of beta-lactam antibiotics through the porin channels of *Escherichia coli* K-12. Antimicrob Agents Chemother.

